# Sensory gating and suppression of subjective peripheral sensations during voluntary muscle contraction

**DOI:** 10.1186/s12868-020-00592-2

**Published:** 2020-10-01

**Authors:** Terumasa Takahara, Hidetaka Yamaguchi, Kazutoshi Seki, Sho Onodera

**Affiliations:** 1 Department of Sport Social Management, KIBI International University, 8 Igamachi, Takahashi, Okayama 716-8508 Japan; 2 Department of Human Health and Wellbeing, University of Marketing and Distribution Science, Kobe. 3-1 Gakuen-Nishimachi, Nishi-ku, Kobe, Hyogo 651-2188 Japan; 3 Department of Health and Sports Science, Kawasaki University of Medical Welfare, 288 Matsushima, Kurashiki, Okayama 701-0193 Japan

**Keywords:** Subjective peripheral sensation, F-wave, Somatosensory evoked potential, Muscle contraction

## Abstract

**Background:**

During voluntary muscle contraction, sensory information induced by electrostimulation of the nerves supplying the contracting muscle is inhibited and the somatosensory evoked potentials (SEPs) amplitude decreases. This depression of sensory input during voluntary muscle contraction has been demonstrated by many studies using electrophysiological methods. However, the association between the electrophysiological response of the sensory system during sustained muscle contraction and subjective peripheral sensation (SPS) is still unclear. The aim of this study was to investigate changes in spinal excitability, SEPs, and SPS during voluntary muscle contraction.

**Results:**

The appearance rate of the F-wave was significantly higher during muscle contraction than rest, whereas no significant difference was observed in F-wave latency between muscle contraction and rest. Furthermore, the P25 amplitude of SEPs was significantly lower during muscle contraction than rest, whereas the N20 amplitude of SEPs exhibited no significant differences. The SPS was significantly lower during muscle contraction than rest

**Conclusions:**

We conclude that sensory gating, which is found in the P25 component of SEPs during muscle contraction, is one of the neurophysiological mechanisms underlying the suppression of SPS.

## Background

Human motor control results from integration of the sensory and motor systems, which are regulated by the relationship between the upper and lower centers, i.e., the brain and spinal cord, respectively. When peripheral nerves are electrically stimulated, a direct motor response (M-wave) occurs in the muscle due to anterograde conduction from the stimulated area. At the same time, the ascending afferent input is projected to the somatosensory cerebral cortex via the spinal cord, and a somatosensory evoked potentials (SEPs) in the cortex can be recorded. During voluntary muscle contraction, sensory information induced by electrostimulation of the nerves supplying the contracting muscle is inhibited and the SEP amplitude decreases [1–4]. The suppression of the sensory potential is known as “gating”. With respect to gating during voluntary movement, previous studies reported that the amount of gating depends on the difficulty of the movement [5], and that gating is observed not only during motion but also pre-motion [6]. Furthermore, suppression of sensory input has already occurred when the spinal cord (a midpoint between the cerebral cortex and periphery) receives input [7]. Therefore, it seems that the main functional role of gating is to eliminate unnecessary sensory information during the execution of purposeful voluntary movements.

F-waves are muscle action potentials recorded when electrostimulation to a peripheral nerve causes retrograde conduction to the axon of an α-motoneuron, and anterograde conduction occurs once again through the automatic firing of a motoneuron in the anterior horn of the spinal cord. The F-wave appearance rate indicates the number of motor units participating in the waveform [8] and naturally varies at a certain width even in the resting state. This natural variation suggests that sensory input influences the effect the central nervous system has on α-motoneurons. H-reflex is also a technique for non-invasive technique which is used to assess motor units activated by the afferent pathway [9]. Previous study using the H-reflex have reported that the excitability of spinal α-motoneurons that innervate active muscles increases during voluntary contraction in the muscle groups of the upper and lower limbs [10]. Furthermore, it is known that the excitability of spinal α-motoneurons increases during remote [11] and contralateral muscle contraction [12]. Therefore, the facilitation of spinal α-motoneurons during muscle contraction may occur not only locally but also systemically.

The depression of sensory input during voluntary muscle contraction has been demonstrated by many studies using electrophysiological methods for animals and humans. It has been reported that P25 amplitude was attenuated but N20 amplitude did not change during voluntary muscle contraction task [1, 13] and reaction tasks [14]. Furthermore, these responses are also observed after the tasks [15, 16]. Thus, based on the result of previous report on the change in each component of SEPs under various conditions, functional significance of attenuation of sensory input during motion is understood. However, the association between the electrophysiological response of the sensory system during exercise and subjective peripheral sensation (SPS) is still unclear. For example, it is often observed that athletes who have pain in the motor system (e.g., mild ankle sprains or muscle soreness) hardly recognize the pain sensation after a few minutes of exercise. However, the neurophysiological aspect of this phenomenon has not been studied to date. Therefore, we hypothesized that the change in electrophysiological response in sensory input affects the subjective peripheral sensation during motor output. Specifically, it is speculated that a decrease in the SPS and SEPs, and an increase in the F-wave appearance rate are observed during motor output such as muscle contraction. The aim of this study was to investigate the changes in spinal excitability, SEPs, and SPS during voluntary muscle contraction.

## Results

**Fig. 1:**
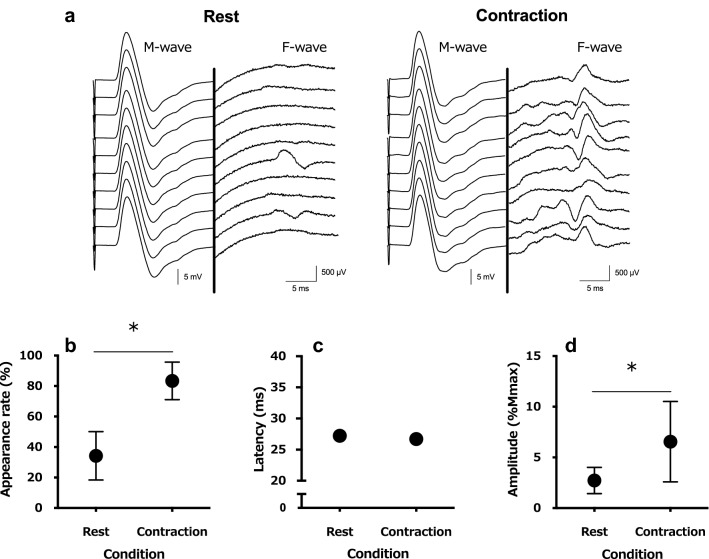
**a** A typical example of an induced electromyogram waveform from a single participant in both conditions (for 10 stimulations). The waveforms of both conditions are M-waves on the left side and F-waves on the right side of the central thick line. The display range of the F-wave is 10 × that of the M-wave. The changes in average F-wave appearance rate (**b**), latency (**c**), and amplitude (**d**) of all participants in both conditions are displayed. *P < 0.05

**Fig. 2:**
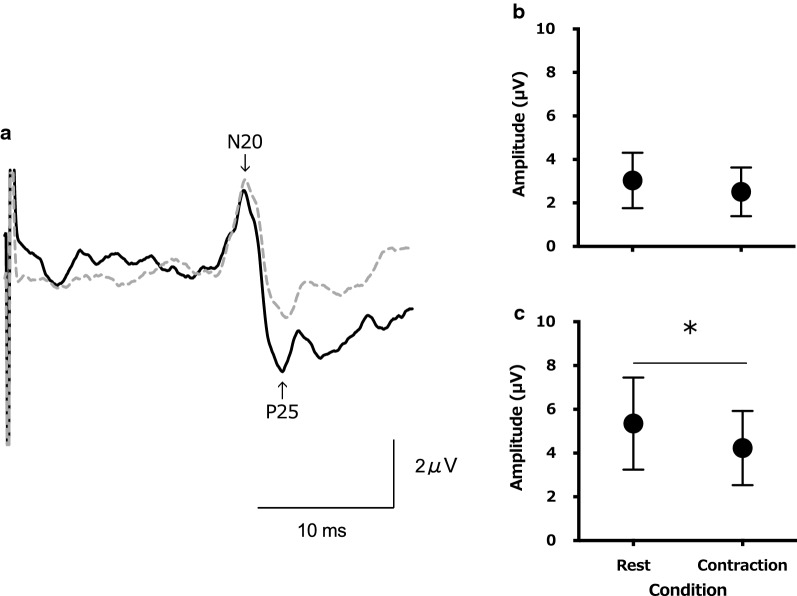
**a** A typical example of a somatosensory evoked potentials (SEPs) waveforms from a single participant (for 200 stimulations) during rest (*solid black line*) and contraction (*gray dashed line*). **b**, **c** The changes in average SEP amplitude for N20 (**b**) and N20-P25 (**c**) of all participants in both conditions. *P < 0.05

**Fig. 3:**
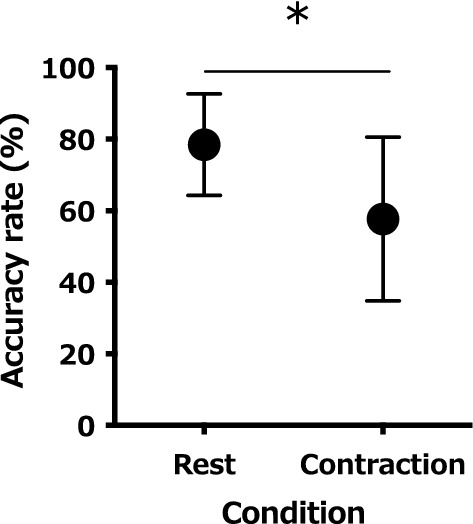
The changes in the average accuracy rate of all participants in both conditions. *P < 0.05

The appearance rate of the F-wave was significantly higher during muscle contraction (83.4 ± 12.3%) compared to rest (34.2 ± 16.0%; t (12) = 9.638, *P* < 0.001; Fig. 1b). No significant differences in F-wave latency were observed between muscle contraction (26.7 ± 1.7 ms) and rest (27.2 ± 1.8 ms; t (12) = 1.257, *P* = 0.23; Fig. 1c). The F-wave amplitude was significantly higher during muscle contraction (6.6 ± 4.0%) than rest (2.7 ± 1.3%; t (12) = 3.661, *P* = 0.003; Fig. 1d). For the N20 amplitude of SEPs, no significant differences were observed between muscle contraction (2.5 ± 1.1 μV) and rest (3.0 ± 1.3 μV; t (12) = 2.083, *P* = 0.06; Fig. 2b). The P25 amplitude of SEPs was significantly lower during muscle contraction (4.23 ± 1.70 μV) compared to rest (5.35 ± 2.10 μV; t (12) = 2.982, *P* = 0.001; Fig. 2c). The accuracy rate of 20 filament stimulations was significantly lower during muscle contraction (57.7 ± 22.9%) compared to rest (78.5 ± 14.2%; t (12) = 4.158, *P* = 0.001; Fig. 3).

## Discussion

In this study, we aimed to investigate changes in spinal excitability, SEPs, and SPS during voluntary muscle contraction. We found that the F-wave appearance rate increased during the isometric pinching task and demonstrated that this natural variation increases during the pinching task. It is possible that the increase in the F-wave appearance rate during the pinching task is caused by suppression of the corticospinal tract, which converges on the spinal anterior motor nerve, and inhibitory systems such as the higher control system.

The potential source of N20 is considered to be the 3b area, which is interpreted simply as the stage at which sensory stimulation has reached the primary sensory cortex via the thalamus [17]. Furthermore, P25 is considered a component derived from a higher level than the 3b area [18]. This suggests that the submaximal isometric pinching force used in our current study suppresses the somatosensory input of a higher level than the 3b area. Previous studies on SEP gating during voluntary movement have reported that gating does not occur in the components corresponding to N20 [19, 20]. For these reasons, although the electrophysiological input that is projected to the primary somatosensory area is the same for a given amount of physical stimulation (regardless of the presence or absence of the motor task), this electrophysiological input is suppressed during the subsequent more complex process of information processing.

In this study, the accuracy rate for cutaneous stimulation at the sensory threshold was used as an index of SPS, and this parameter was reduced by submaximal isometric muscle contraction. This is considered the result of presynaptic inhibition of peripheral sensation during motor output. During muscle contraction, some sensory inputs derived from skin sensory receptors are already suppressed within peripheral nerves, i.e., before reaching the neural circuits of the brain and spinal cord [21]. The numerous neural circuits in the cerebral cortex and spinal cord play various important roles during motor behavior. The results of this study suggest that there is a mechanism that facilitates the necessary circuits by inhibiting unnecessary circuits that are related to motion during motor output.

Short latency (P27-N20, and N33-P27) SEP components were decreased after transcranial focused ultrasound (tFUS) to S1 that also enhanced performance on sensory discrimination tasks [22]. With respect to these results, the authors hypothesized that the pulsed acoustic pressure wave to primary sensory cortex (S1) may locally shift the balance of excitation and inhibition by acting on mechanically sensitive components of the brain (cell membranes, ion channels and synaptic vesicle cycles). Based on this hypothesis, it was explained that the pulsed acoustic pressure waves dampen excitation or increase local interneuron firing led to attenuation of SEP amplitudes, resulting in an improvement in cortical representation of tactile stimuli. Further, S1 is already activated by the input from primary motor cortex (M1) before receiving the input from the sensory receptors during voluntary movement [23]. In the present study as well, it can be predicted that S1 was activated with the execution of the muscle contraction task. However, since the input from the peripheral sensory receptor to S1 is hardly necessary in the isometric muscle contraction task at the submaximal strength as in the present study. Therefore, it is considered that the inhibitory adjustments for S1 activated by the input from M1 was occurred. Inhibitory effect on the S1 seems to contribute to reduce the sensitivity of peripheral sensation. This hypothesis helps to explain the decrease in P25 amplitude in the present study.

Furthermore, it is possible that the threshold of cutaneous surface sensation was increased by the motor output. Most of the decrease in the accuracy rate observed in this study was due to the increased inability to recognize the sensory stimulation. This indicates the need for a test in which participants could recognize sensory information correctly. There was a certain degree of freedom regarding the stimulation site and filament stimulation intensity, as the sensory stimulation was applied manually in this study. This implies that the sensory threshold during motor output does not increase uniformly at all sites but varies according to each site or receptor.

Findings of this study suggest the neurophysiological aspect of changes in subjective sensation during muscle contraction and that subjective sensations in humans are closely related with the attenuation of sensory potential.

## Conclusions

The present study has demonstrated that increasing spinal excitability, which innervates active muscle groups, causes depression of P25 components in the operating limb area that governs muscle group actions. As such, we have clarified the mechanisms underlying SPS of operating limbs. We conclude that sensory gating, which is found in the P25 component of SEPs during muscle contraction, is one of the neurophysiological mechanisms responsible for suppressing SPS.

## Methods

### Participants

Participants comprised 13 healthy adult males with no history of neurophysiological diseases. Mean (± SD) age was 27 ± 11 years, height was 167 ± 2 cm, and weight was 66 ± 6 kg). This study was conducted in accordance with the Declaration of Helsinki. Informed consent to participate in the study was obtained from the participants verbally and in writing. This study was approved by the Ethical Review Board of the Kibi International University (No. 18-37).

### Electrical stimulation

A square wave pulse lasting 0.2 ms was generated in the median nerve in the carpal area on the right side (i.e., ipsilateral to the pinching hand). Using a surface electrostimulation apparatus (NM-420S, Nihon Kohden, Japan), the stimulation electrodes were placed 20 mm apart, with the cathode proximal and the anode distal. The stimulation electrodes were secured using a fixation band to regulate their pressure. The minimum intensity of electrostimulation to induce an M-wave was confirmed, and the stimulation region was set appropriately.

### F-wave recording and analysis

A surface electromyogram (EMG) during electrostimulation was recorded in the right abductor pollicis brevis using Ag/AgCl bipolar electrodes (5 mm diameter, 20 mm interelectrode distance; Nihon Kohden, Japan) on the same side as the electrostimulation. The recording electrode was attached onto the muscle belly of the abductor pollicis brevis, and the reference electrode onto the first proximal phalanx; both electrodes were fixed with surgical tape. The attachment site for the recording electrode was wiped with alcohol and preprocessed using sandpaper to create an interelectrode resistance < 5 kΩ. An EMG/evoked potential testing device (Neuropack MEB-9404, Nihon Kohden, Japan) was used, and F-wave waveforms were recorded using a band-pass filter of 1.5 − 3 kHz and a sampling frequency of 10 kHz. The electrostimulation intensity was set to 1.2x, which obtained a maximum M-wave. The stimulation frequency was set to 1 Hz and was carried out for approximately 1 min. The F-wave analysis used the three factors appearance rate, latency, and amplitude F/M ratio during the second half (30 s) of electrostimulation. To determine the percentage of the appearance rate, all identifiable resulting waveforms (total n = 30) on the monitor (500 μV/D) were targeted. For the F-wave amplitude, the average value of the peak-to-peak amplitude for the F-waves was expressed as a ratio compared to the maximum M-wave amplitude. Latency was the average time from electrostimulation until F-wave initiation.

### SEP recordings and analysis

Based on the international 10–20 system, the SEPs were recorded from the somatosensory area of the right upper arm (C3′: 2 cm posterior from C3) on the stimulation side. The reference electrode was placed at the point Fz. Electrodes were attached onto the skin surface using conductive paste. Interelectrode resistance was set to < 5 kΩ. The electrostimulation intensity was fixed at just above the motor threshold with a repetition rate of 3 Hz. An EMG/evoked potential testing device (Neuropack MEB-9404, Nihon Kohden, Japan) was used, and SEP waveforms were recorded using a band-pass filter of 20 Hz − 10 kHz and a sampling frequency of 10 kHz, and 200 responses were averaged. SEP waveforms were evaluated for 100 ms, evaluations being performed at the time of electrostimulation and 100 ms after stimulation. Epochs with artifacts due to eye movement or blinking (> ± 6 μV from baseline) were excluded automatically prior to averaging. A plate electrode was used to record the evoked electroencephalogram (Ag/AgCl electrode, NE-132B (Φ 10 mm), Nihon Kohden, Japan). The amplitudes from base line to N20, and N20-P25 peak-to-peak amplitude, which are early components after electrostimulation, were analyzed for the SEPs. The amplitude of each component was measure from preceding peaks.

### Subjective peripheral sensation (SPS)

Prior to the experiment, the SPS threshold on the dorsal surface of the right hand was measured using a Semmes–Weinstein monofilament test (SAKAI Medical Co., Ltd., Tokyo, Japan). After establishing their peripheral sensory thresholds, participants reported the presence or absence of peripheral cutaneous stimulation during the SPS measurements. Monofilament stimulation was carried out using gradually thicker filaments, starting from thin filaments of 0.008 g. Confirmation tests were repeated approximately five times for each intensity, and the filament thickness that could be sensed correctly at a rate of approximately 100% was defined as the SPS threshold. The experimenter lowered the filament vertically onto the hand, removed it, and returned it to its original position within 1 s. The stimulation interval of the filament was random and carried out a total of 20 times. The participants were instructed to give verbal cues when they sensed filament stimulation; afterwards, their accuracy rate was calculated. All measurements were carried out by the same experimenter. The filament stimulation site was marked with a marker, so that deviation of a large stimulation site due to the experimenter error does not occur.

### Testing procedure

The participants were seated in a chair with both arms placed on armrests with open eyes to perform a pinching task with the right hand while keeping the arm in a neutral position. Before the task, maximum voluntary isometric force (MVIF) was measured, from which the target force level was calculated. The participants held the pinch force meter with the thumb and index finger, and performed the force exerting with all one's might for 5 s in the experimental position. The maximum value at the time of force exertion of 5 s was defined as MVIF. The target value was displayed visually on a computer placed 1 m in front of the participant. After sufficient rest of more than 15 min, participants maintained 30% MVIF for approximately 2 min. Force was not exerted in the rest condition. Each measurement item and each condition were randomly set and measured for each subject.

### Statistics

All values are shown as the mean ± standard deviation. Comparisons were analyzed using paired t-tests; statistical significance levels were set at < 5% (*P* < 0.05) with GraphPad Prism Ver 8.3.1 for Macintosh (GraphPad Software, San Diego, CA, USA).

## Data Availability

The datasets supporting the conclusions of this article are included within the article (and its additional files).

## Abbreviations

SEPs: Somatosensory evoked potentials
SPS: Subjective peripheral sensation
EMG: Electromyogram
MVIF: Maximum voluntary isometric force
